# A discriminative method for family-based protein remote homology detection that combines inductive logic programming and propositional models

**DOI:** 10.1186/1471-2105-12-83

**Published:** 2011-03-23

**Authors:** Juliana S Bernardes, Alessandra Carbone, Gerson Zaverucha

**Affiliations:** 1 COPPE, Programa de Engenharia de Sistemas e Computação, Universidade Federal do Rio de Janeiro, Rio de Janeiro, Brazil; 2 Université Pierre et Marie Curie, UMR7238, Génomique Analytique, 15 rue de l'Ecole de Médecine, F-75006 Paris, France; 3 CNRS, UMR7238, Laboratoire de Génomique des Microorganismes, F-75006 Paris, France

## Abstract

**Background:**

Remote homology detection is a hard computational problem. Most approaches have trained computational models by using either full protein sequences or multiple sequence alignments (MSA), including all positions. However, when we deal with proteins in the "twilight zone" we can observe that only some segments of sequences (motifs) are conserved. We introduce a novel logical representation that allows us to represent physico-chemical properties of sequences, conserved amino acid positions and conserved physico-chemical positions in the MSA. From this, Inductive Logic Programming (ILP) finds the most frequent patterns (motifs) and uses them to train propositional models, such as decision trees and support vector machines (SVM).

**Results:**

We use the SCOP database to perform our experiments by evaluating protein recognition within the same superfamily. Our results show that our methodology when using SVM performs significantly better than some of the state of the art methods, and comparable to other. However, our method provides a comprehensible set of logical rules that can help to understand what determines a protein function.

**Conclusions:**

The strategy of selecting only the most frequent patterns is effective for the remote homology detection. This is possible through a suitable first-order logical representation of homologous properties, and through a set of frequent patterns, found by an ILP system, that summarizes essential features of protein functions.

## Background

An important problem in Computational Biology is the detection of remote homologous proteins, that is, proteins that have a common ancestor but that have diverged significantly in their primary sequence in evolutionary history. In practical terms, remote homology detection is the problem of detecting homology in cases of low sequence identity, frequently below 30%. This is an important and hard problem, thus the development of methods to identify homologs between proteins is essential for functional and comparative genomics. In a general way, homology detection methods are today very important to help for sequence annotation and to guide laboratory experiments.

Traditional methods, such as BLAST [1], deal with the homology detection problem by searching for regions of local similarity among pairs of sequences. Certainly, the performance of these methods is directly related to sequence identity, and since remote homologous sequences have low identity, those methods fail to give satisfiable answers. An alternative to BLAST are generative methods. First, they train a model to represent a group of homologous sequences (a protein family), and then match a query sequence against the model to evaluate the similarity of the query sequence to the group/family. Profile Hidden Markov Models (pHMMs) [2] and PSI-BLAST are examples of such approaches, also known as family-based or sequence-profile based approaches. PSI-BLAST builds a probabilistic model based on Position Specific Score Matrix (PSSM) from the results of the first BLAST alignment. Next, this PSSM is used to further search on the database for new matches, and is updated for subsequent iterations with newly detected sequences. Several studies have shown that sequence-profile based approaches perform better than methods based on pairwise comparison only [3], but they still largely fail to detect distant homologous proteins. A significant improvement over those methods were made possible by comparing profile-profile instead of sequence-profile. Methods such as PROF-SIM [4], COMPASS [5] and HHsearch [6] build a profile from the query protein and then compare it against a profile database constructed from the target proteins.

The performance of generative methods degrades as sequence similarity decreases. This limitation has motivated researchers mainly in two directions, i) to combine extra information to previous approaches, such as phylogenetic [7] and protein structure information [8,9], and ii) to search for new accurate methods. Among the new approaches, a family of methods called "discriminative", have been able to attain additional accuracy to remote homology detection by modeling the differences between positive and negative examples. The most popular discriminative method applied to the remote homology detection problem is Support Vector Machine (SVM) [10-26]. Basically, SVMs learn a classification function, from positive and negative training examples, that optimally separates the unseen data into two categories, for instance, homologous and non-homologous proteins. The kernel function that measures the similarity between a pair of examples has a key role in the SVM performance. Typical approaches represent each protein sequence as a fixed-length vector, where each vector's item is a protein property, and design a kernel function taking the inner product between these vectors. Several feature protein vector representations have been proposed. SVM-Fisher [10] represents each protein *x *as a vector of Fisher scores. These scores are obtained comparing *x *to the pHMM built from the positive training sequences (a protein family). SVM-pairwise [14] also uses scores to compose its feature vector, those are extracted from pairwise alignments of *x *and each sequence in the training set. Some methods use representations based on primary-sequence motifs, where a sequence *x *is represented in a vector space indexed by a set of precomputed motifs [11]. GPkernel [23] is another method based on motifs, but instead of using precomputed motifs coming from an existing database, it generates motifs from training data. Other methods have used structural motifs in place of primary-sequence ones for feature extraction task. The SVM-I-sites method [12] constructs the feature vector of a protein *x *by comparing the *x *profile (built by using PSI-BLAST) to the I-sites library of local structural motifs. Later, this work was improved taking into account the order and relationship of the I-sites motifs [15]. A series of works have explored the use of k-mers (short subsequences of size k). Mismatch kernel [13] represents a sequence *x *as a vector of k-mers occurrence, that is, each vector position has a non-zero weight if the k-mer is present in *x *and zero weight otherwise. A k-mer is said to be present in *x *if *x *contains a substring that has at most n mismatches to the k-mer. Profile kernel [19] vector representation is similar to Mismatch kernel one. However, it considers a k-mer to be present if *x *contains a substring whose PSSM-based ungapped alignment scores with the k-mer is above an user defined threshold. A feature vector representation based on distances between k-mers was introduced in [21]. Statistical and relevant features have been extracted from all possible k-mers (coming from training protein sequences) by using latent semantic analysis (an efficient feature extraction technique from natural language processing) in [22]. Later, this work was improved by using Top-n-grams that are extracted from protein sequence frequency profiles [24].

On the other hand, some approaches have followed a way that is alternative to the feature protein vector representation by pre-computing a kernel matrix where each element is the measure of similarity between a pair of examples. This matrix can be used directly as a kernel function. Some new tools have followed this way, such as SVM-LA [16], which measures the similarity between a pair of sequences by summing up scores obtained from its local alignment, and SW-PSSM [20], which uses profiles scoring schemes for measuring the similarity between pairs.

Most of SVMs are family-based, that is, a protein family is required to train them, and the aim is to classify unseen proteins as member or non-member of this family. Certainly, these methods are limited to the number of known families. In order to overcome this drawback, a new SVM category has been proposed, that is, pairwise SVM [25,26]. Here, the aim is to rank proteins that are homologs to a given query protein. These methods are an alternative to the most commonly used methods in the biology community, that is: BLAST and PSI-BLAST.

In fact, in the SVM-HUSTLE [25] a training strategy was presented that could convert a family-based SVM into a pairwise SVM. Like PSI-BLAST it iteratively searches for homologs against a database by using BLAST in the first iteration. Next, it trains concurrent SVMs from positive sequences selected from BLAST output, and negative sequences selected randomly from the remaining database. Then, trained SVMs scan the database searching for new homologs that are added to the positive set. The algorithm stops when no new sample is classified as positive or when a maximum number of iterations is achieved.

To improve the performance some methods have been applied the semi-supervised training strategy, that is, they combine information from labeled and unlabeled databases in order to recruit more training sequences. This strategy is generally applied when unlabeled data is abundant while labeled data is limited, and this is the actual scenario of protein classification, since there is a large group of still unannotated proteins. However, semi-supervised approaches can become computationally hard when unlabeled large databases are used, such as nrdb90. Among methods that employ this strategy are RANKPROP [27], SVM-HUSTLE [25], Top-N-Gram [24] and SW-PSSM [20].

SVM methods are among the most effective and accurate methods for solving the remote homology detection problem. They classify query sequences as member or non-member of homologous proteins, but they do not provide any insight to the user concerning the reasons for the separation. Moreover, SVM is not able to work directly over relational data. However, biological data is naturally relational. For example, a specific amino acid in a protein could belong to an α-helix and at the same time belong to the active site of that protein. Therefore, methodologies that explore relational data are expected to be more suitable to deal with biological data. In this vein, we focus our attention on Inductive Logic Programming (ILP) [28]. ILP is a relational data-mining method that uses first-order logic predicates to represent background knowledge, theories and examples (positives and negatives). From those an ILP system can learn a hypothetical logic program which entails all the positive and none of the negative examples. This logic program is a comprehensible set of logical rules that can be used to classify unseen examples. Moreover, when applied to remote homology detection problem, it can provide insights into conserved features of homologous protein families.

To the best of our knowledge, researchers have developed two approaches for applying ILP to remote homology detection. The first method is known as *Homology Induction *[29,30] and uses ILP to improve on conventional sequence-based homology method. The second method uses a hybrid ILP-propositional machine learning method to predict protein functional classes directly from sequences [31,32]. First, it represents each protein through first-order logic predicates. It creates predicates based on properties extracted directly from sequences, such as frequency distribution of single residues, and on properties predicted from sequences such as secondary structure elements. Second, it uses WARMR [33], an ILP data-mining program, to identify the most frequent patterns in its knowledge base. Third, it converts these frequent patterns into binary attributes to be used in propositional learning. Finally, it uses decision trees (DTs) [34] as propositional machine learning method.

**Figure 1:**
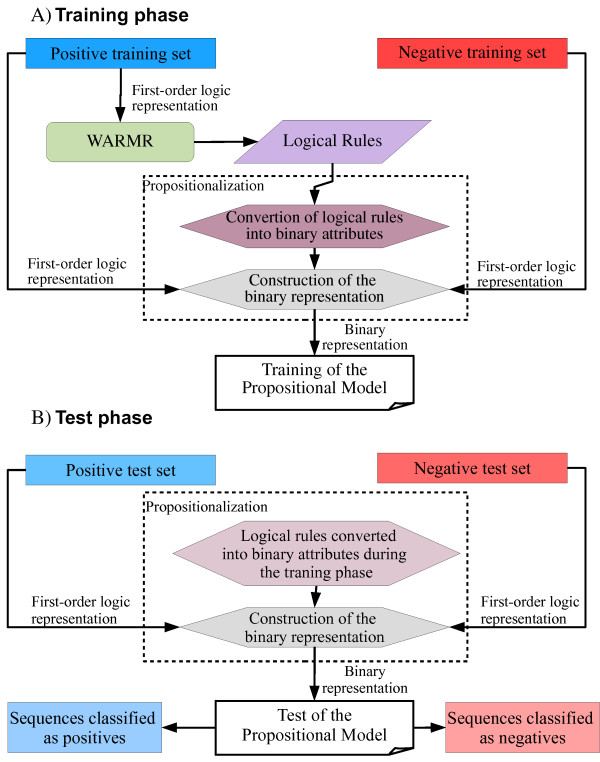
**Flowchart of the method**. A) Training phase. Each sequence in the positive training set is represented through first-order logic predicates. WARMR learns logical rules on the set. These rules are converted into binary attributes in order to train propositional models; this step is called propositionalization. Next, each sequence in the positive and negative training set is represented through binary attributes, and finally propositional models, such as DTs or SVM, are trained. B) Test phase. Each sequence in the positive and negative test set is represented through binary attributes that correspond to the logical rules learned during the training phase. Next, the propositional model is tested and its output is divided into sequences classified as positives and sequences classified as negatives.

Our work is based on the same basic approach [31,32]. However, there are significant differences. First, we have proposed a novel first-order logical representation based on conserved amino acid positions in a multiple sequence alignment (MSA). Second, we have related the first-order logical representation based on sequence properties, proposed in [31,32,35], with our novel representation based on conserved positions for creating a hybrid representation that takes into account conserved physico-chemical positions in a MSA. Third, we have joined features created by these representations to train propositional models. In a general way, this combination of features has improved the performance of models. Fourth, we have proposed to use SVM as propositional machine learning method rather than DTs. Figure 1 summarizes the proposed methodology.

**Figure 2:**
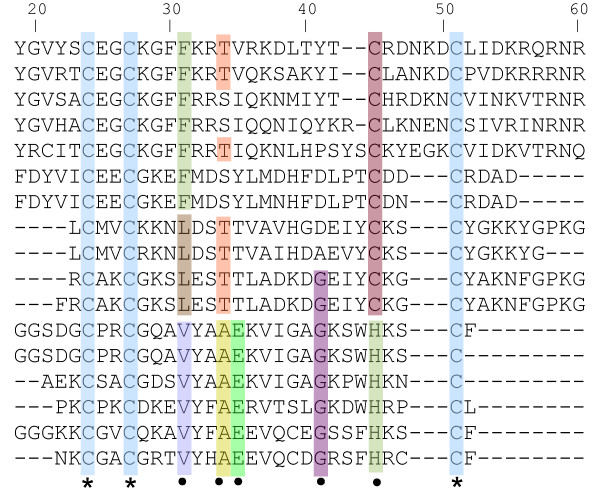
**Partial alignment of "Glucocorticoid receptor-like (DNA-binding domain)" superfamily sequences**. Some conserved positions are highlighted: * marks completely conserved positions, • marks partially conserved positions. The alignment is built by using CLUSTALW.

We confirmed that building models using only the most frequent patterns is a suitable methodology to the remote homology detection problem. Remote homologous proteins seem to share only the essential properties in order to keep their function, and these properties can be represented by first-order logic predicates. For instance, Figure 2 shows the partial alignment of "Glucocorticoid receptor-like (DNA-binding domain)" superfamily sequences. The sequence identity of this alignment is smaller than 30%. We can observe that some positions are completely conserved (marked by *). Also, there are positions which are partially conserved (marked by •). Methods that have the ability of exploring only these positions most likely will outperform the methods that consider the whole alignment, since non-conserved positions could add noise to the model.

Our intention in this paper was to investigate if the performance of remote homology detection methods could be improved only by exploring the most frequent patterns into homologous sequence groups. Therefore, we did not use any extra-information, such as structural properties or phylogenetic trees, contrary to [31,32] that used structural properties. All training data was extracted from sequences and MSAs. We carried out experiments on remote homology benchmarks and showed that SVM outperforms DTs when they are trained by using only the most frequent patterns. Also, we have demonstrated that good performance can be achieved when we used first-order logical representations for the protein sequences based on conserved amino acid positions and based on conserved physico-chemical positions in the MSA. Moreover, we showed that the combination of features created by different first-order logical representations improves the performance of propositional models. Finally, we compared the performance of our models with state of the art methods and showed that they are comparable to or better than its competitors, this includes the cases where sequence identity is low (below 30%). However, the output of our method can be interpreted biologically to provide insights into conserved features of homologous protein families.

## Results and Discussion

**Figure 3:**
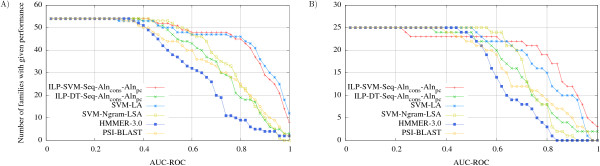
**Performance as measured by AUC-ROC**. For each SCOP family, we run SVM and DT models *T *times (see Methods) over the same positive set, but over several different negative sets. We calculated the AUC-ROC for each run and the AUC-ROC of SCOP family by averaging the AUC-ROC on all runs. We plot the AUC-ROC versus the number of families that achieved a given AUC-ROC score or better for the database *S_full _*(A) and for the database *S_30 _*(B). We shows only ILP models that achieved best performance. We carried out rank-sum test [38] to compare the curves, see Table 4. For both databases ILP-SVM-Seq-Aln**_cons_**-Aln**_ps _**outperformed all methods, except SVM-LA, and ILP-DT-Seq-Aln**_cons_**-Aln**_ps _**outperformed only HMMer-3.0.

In order to assess our methodology, we have trained DTs and SVMs using representations described in Methods. We called *Seq *those models that are trained from sequential properties only, and we named *Aln_cons _*those models that are trained from conserved amino acid positions in a MSA. We have created a hybrid representation that takes into account conserved physico-chemical positions in a MSA (see *R*_6 _in Table 1), the resulting models were called *Aln_pc_*, where *pc *is an abbreviation for physico-chemical properties. Additionally, we created models by joining *Seq*, *Aln_cons _*and *Aln_pc _*features. We named ILP-SVM and ILP-DT models trained from our methodology. Table 2 summarizes results (see also Figure 3 that shows only ILP models with best performance). ILP-DT models did not reach good performance on *S_full _*and *S*_30 _databases (see Methods). ILP-SVM-*Seq*-*Aln_cons_*-*Aln_pc _*models outperformed all other ILP methods for both databases.

**Table 1:** Logical rules constructed by WARMR.

WARMR output
R_1 _: Homologous(A):- col(A,c,24), col(A,c,27), col(A,c,51) **1.0**
R_2 _: Homologous(A):- col(A,c,45) **0.7**
R_3 _: Homologous(A):- col(A,k,29) **0.45**
R_4 _: Homologous(A):- hydrophobic(A,2) **0.7**
R_5 _: Homologous(A):- aminoacidPairRatio(A,cg,1) **0.77**
R_6 _: Homologous(A):- col(A,B,34), small(B) **1.0**
**Interpretation**
R_1 _: 100% of homologous proteins have the C amino acid in positions 24, 27 and 51.
R_2 _: 70% of homologous proteins have the C amino acid in position 45.
R_3 _: 45% of homologous proteins have the K amino acid in position 29.
R_4 _: 70% of homologous proteins have between 10 and 20% of hydrophobic amino acids.
R_5 _: 77% of homologous proteins have at least 1 pair of CG.
R_6 _: 100% of homologous proteins have a small amino acid in positions 34.

Some logical rules learned from "Glucocorticoid receptor-like (DNA-binding domain)" sequences and their alignment (see Figure 2). They are shown in their original WARMR output and their interpretation is given below.

**Table 2:** Average AUC for S_full _and S_30 _databases.

	**S**_**full**_	**S**_**30**_
ILP-SVM-Seq	0.79	0.77

ILP-SVM-Aln**_cons_**	0.81	0.81

ILP-SVM-Aln**_ps_**	0.80	0.81

ILP-SVM-Seq-Aln**_cons_**	0.85	0.80

ILP-SVM-Aln**_cons_**-Aln**_ps_**	0.82	0.82

ILP-SVM-Seq-Aln**_cons_**-Aln**_ps_**	**0.87**	**0.82**

ILP-DT-Seq	0.67	0.65

ILP-DT-Aln**_cons_**	0.70	0.69

ILP-DT-Aln**_ps_**	0.68	0.67

ILP-DT-Seq-Aln**_cons_**	0.72	0.69

ILP-DT-Aln**_cons_**-Aln**_ps_**	0.71	0.71

ILP-DT-Seq-Aln**_cons_**-Aln**_ps_**	0.74	0.71

SVM-Ngram-LSA	0.79	0.77

SVM-LA	**0.87**	0.80

PSI-BLAST	0.75	0.69

HMMer-3.0	0.63	0.60

We highlighted that the novel logical representation, based on conserved amino acid positions (*Aln_cons_*) and based on conserved physico-chemical positions (*Aln_pc_*) in MSA, that we propose here, is able to achieve better prediction accuracy than the sequential logical representation commonly used by related works. This result is expected since it is known that MSAs contain more functional and structural information than properties extracted from unaligned sequences. On the one hand, the combined models (*Seq*-*Aln_cons_*, *Aln_cons_*-*Aln_pc _*and *Seq*-*Aln_cons_*-*Aln_pc_*) were able to attain better accuracy than single models (*Seq*, *Aln_cons _*and *Aln_pc_*) for the *S_full _*database. On the other hand, for the *S*_30 _database, the sequential logical representation does not contribute to improve the model performances. Based on the observation that MSA information is richer than sequential properties, we tested the hypothesis that below 30% of sequence-identity, MSA information is still rich enough to build accurate models and that sequential properties might add noise within combined models. In fact, we observed that ILP-SVM-*Aln_cons _*outperformed ILP-SVM-*Seq*-*Aln_cons_*, while ILP-SVM-*Seq*-*Aln_cons_*-*Aln_pc _*and ILP-SVM-*Aln_cons_*-*Aln_pc _*achieved a similar perfomance.

When we compare ILP-SVM models with ILP-DT models, all ILP-SVM models outperformed ILP-DTs. In fact, SVMs are often more accurate than DTs. We observed that ILP-DTs have produced fewer and simpler rules for both databases and they presented a poorer classification on test examples. In order to provide a comparison with [31,32], we considered for comparison the ILP-DT-Seq model, since it uses all properties handled in [31,32], except those predicted from sequences, such as secondary structure. Our results show that all models proposed here outperformed ILP-DT-Seq for both databases.

When we combine the representations the number of features can increase creating sparse data. However, this can be avoided by using a feature selection technique. Here, we applied chi-square statistical test to remove class uncorrelated rules. We set *δ *for 0.05 and 0.25, see Methods. Table 3 shows how AUC values vary according to and without the chi-square test. We can observe that for most methods the performance was kept with *δ *= 0.05 with a significant reduction in the number of features. On the other hand, *δ *= 0.25 worsened the performance for all methods. In fact, for some families *δ *= 0.25 removed all logical rules.

**Table 3:** Average AUC and number of logical rules according to chi-square test for S_full _and S_30 _databases.

Methods	*S_full_* AUC (#logical rules)			S_30_ AUC (#logical rules)		
	
	no chi-square	*δ *= 0.05	*δ *= 0.25	no chi-square	*δ *= 0.5	*δ *= 0.25
ILP-SVM-Seq	0.79 (228.59)	0.79 (89.15)	0.75 (59.30)	0.77 (311.09)	0.77 (91.04)	0.70 (26.4)

ILP-SVM-Aln**_cons_**	0.81 (44.91)	0.81 (34.98)	0.77 (12.76)	0.81 (56.72)	0.80 (36.44)	0.72 (13.16)

ILP-SVM-Aln**_ps_**	0.80 (191.65)	0.79 (139.61)	0.75 (66.15)	0.81 (241.96)	0.81 (178.72)	0.73 (71)

ILP-SVM-Seq-Aln**_cons_**	0.85 (311.09)	0.83 (144.07)	0.79 (35.8)	0.80 (381.12)	0.80 (178.56)	0.74 (49.96)

ILP-SVM-Aln**_cons_**-Aln**_ps_**	0.82 (236.56)	0.82 (174.59)	0.79 (46.04)	0.82 (283.12)	0.82 (209.76)	0.80 (57.28)

ILP-SVM-Seq-Aln**_cons_**-Aln**_ps_**	0.87 (502.74)	0.85 (283.69)	0.81 (74.3)	0.82 (623.56)	0.82 (357.28)	0.79 (90.96)

ILP-DT-Seq	0.67 (228.59)	0.67 (89.15)	0.62 (59.30)	0.65 (311.09)	0.65 (91.04)	0.61 (26.4)

ILP-DT-Aln**_cons_**	0.70 (44.91)	0.70 (34.98)	0.72 (12.76)	0.69 (56.72)	0.69 (36.44)	0.65 (13.16)

ILP-DT-Aln**_ps_**	0.68 (191.65)	0.68 (139.61)	0.64 (66.15)	0.67 (241.96)	0.67 (178.72)	0.62 (71)

ILP-DT-Seq-Aln**_cons_**	0.72 (311.09)	0.71 (144.07)	0.67 (35.8)	0.69 (381.12)	0.68 (178.56)	0.63 (49.96)

ILP-DT-Aln**_cons_**-Aln**_ps_**	0.71 (236.56)	0.71 (174.59)	0.73 (46.04)	0.71 (283.12)	0.70 (209.76)	0.62 (57.28)

ILP-DT-Seq-Aln**_cons_**-Aln**_ps_**	0.74 (502.74)	0.74 (283.69)	0.69 (74.3)	0.71 (623.56)	0.71 (357.28)	0.63 (90.96)

We compared our best models, that is, those trained from *Seq*-*Aln_cons_*-*Aln_pc _*features, with two models based on SVM (SVM-Ngram-LSA [22] and SVM-LA [16]), and also with two other widely used methods: HMMer-3.0 [36] and PSI-BLAST [37]. We carried out rank-sum tests [38] to compare models listed in Figure 3, and we show in Table 4 statistical measures for this comparison. For both databases, ILP-SVM-*Seq*-*Aln_cons_*-*Aln_pc _*outperformed SVM-Ngram-LSA, PSI-BLAST and HMMer-3.0, and achieved comparable performance to SVM-LA. ILP-DT-*Seq*-*Aln_cons_*-*Aln_pc _*model achieved better results than HMMer-3.0, and achieved similar performance to PSI-BLAST. Based on ILP-SVM-*Seq*-*Aln_cons_*-*Aln_pc _*performance, we can conclude that the combination of alignment information and sequence properties, and the strategy of selecting only the most important features can yield a more accurate model than those that explore all alignment positions, as HMMer-3.0 and PSI-BLAST, and those that extract Ngram from unaligned sequences, such as SVM-Ngram-LSA.

**Table 4:** Rank-sum test p-values for curves of Figure 3.

*S_full_*				
	**SVM-LA**	**SVM-Ngram-LSA**	**PSI-BLAST**	**HMMer-3.0**

ILP-SVM-Seq-Aln**_cons_**-Aln**_ps_**	0.93	4.93e-05	2.55e-07	5.63e-07

ILP-DT-Seq-Aln**_cons_**-Aln**_ps_**	7.27e-06	2.23e-04	0.17	5.44e-07

***S*_30_**				

ILP-SVM-Seq-Aln**_cons_**-Aln**_ps_**	0.07	0.05	3.85e-05	1.33e-06

ILP-DT-Seq-Aln**_cons_**-Aln**_ps_**	1.44e-05	0.015	0.41	1.2e-05

We consider a result with p ≤ 0.05 to be significant.

Although the results show that ILP-SVM-*Seq*-*Aln_cons_*-*Aln_pc _*outperformed some state-of-art methods, we emphasize that the performance of PSI-BLAST depends on the number of iterations and on the size of the database used to build the profiles. Thus, to extract the maximum performance of PSI-BLAST, we adopt the semi-supervised training strategy and we used nrdb90 as unlabeled database and set the number of iterations to 20, as done in [26]. Unsurprisingly, it performed better than our ILP-SVM models. For example, PSI-BLAST_*nr*20 _achieved a AUC of 0.88 for the database *S_full _*and 0.83 for the database *S_30_*. Certainly, methods trained from the nrdb90 database are expected to build more accurate models and be more effective in annotating remote homologous proteins. However, the computation time of methods that adopt semi-supervised training depends on size of the unlabeled database. Therefore, PSI-BLAST run on this configuration is computational time consuming. In conclusion, when supervised training strategy is employed ILP-SVM methods obtain better or comparable performance than its competitors.

In order to provide an example of the biological interpretation of the logical rules constructed by WARMR, we show in Table 1 some rules which have been learned on members of the "Glucocorticoid receptor-like (DNA-binding domain)" superfamily. Rules R_1_, R_2 _and R_3 _were learned from conserved amino acid positions (*Aln_cons_*) and R_6 _from conserved physico-chemical positions (*Aln_pc_*), see Figure 2, while rules R_4 _and R_5 _were learned from sequential properties. These rules represent only the conserved properties of "Glucocorticoid receptor-like (DNA-binding domain)" superfamily members, that is, these rules catched essential features identifying the superfamily members. This was possible by using first-order logic predicates to represent the properties of each superfamily member, and by applying ILP in order to filter the essential features.

## Conclusions

We have combined ILP and propositional models for improving the accuracy of remote homology detection methods. Our approach can be segmented into three parts. First, training sequences are represented through first-order logic predicates. Similar to [31,32,35], we have used a representation based on sequence properties. Additionally, we introduced a novel representation based on conserved amino acid positions in protein alignments. Also, we related the logical representation based on sequential properties with our logical representation based on conserved positions creating a new representation for conserved physico-chemical positions in a MSA. Second, we executed WARMR, an ILP system, in order to find only the most frequent patterns in our training set. Third, the logical rules learned in the previous stage were converted in binary attributes for training propositional models. Here, we applied decision trees and the widely used support vector machine as propositional methods.

Our methodology is partly similar to the study developed in [31,32]. However, we proposed a novel logical sequence representation based on conserved positions in MSA; we combined this representation with the logical representation based on sequence properties only, proposed in [31,32,35]; we applied SVMs rather than DTs. We showed that the prediction performance of our method, that uses logical representation of alignment information, is better than the prediction performance of our models trained only on the sequential representation. Also, the combined representations improved the performance of ILP-DT models in any sequence identity range and the performance of ILP-SVM for the original database. We carried out comparisons among the models proposed here with models based on SVM (SVM-Ngram-LSA and SVM-LA), a model closer to the model proposed in [31,32], that is, ILP-SVM-Seq, HMMer-3.0 and PSI-BLAST. Our experiments showed that for the same data set, ILP-SVM models achieves a superior or a comparable performance for any sequence identity range. In particular, our method produces a human-understandable output that can provide insights about conserved features of protein families.

We can conclude that the first-order logic language is suitable to represent conserved protein properties, and that from this representation, an ILP system can learn the essential rules that discriminate between homologous and non-homologous proteins. Our methodology supports the intuition that proteins with remote evolutionary relationship have suffered several mutational events, and that only essential amino acids and their physico-chemical properties are kept in evolved sequences. Thus, computational methods that explore only the conserved positions are more suitable to the remote homology detection problem than the methods that explore all amino acids within sequences.

We have confirmed through this study that conserved alignment positions play an important role in recognizing remote homologous proteins contrary to sequential properties extracted from unaligned sequences. We highlight that sequential properties can be useful for helping to identify remote homologous proteins, however, when the sequence identity is smaller than 30%, this information might become noise and worsens the performance of methods, as we observed for ILP-SVM-Seq-*Aln_cons_*.

Another advantage of our methodology is the simplicity to include additional sequence properties. For this we can create a new predicate that represents the property and no modification of the algorithm is necessary. In this study, we used only properties that can be extracted directly from sequences or from conserved alignment positions. We considered a limited number of amino acid physico-chemical properties (only 16), since our logic sequential representation is based on previous ones [31,32]. However, the Amino Acid Index Database [39] has defined amino acid numerical indices for more than 500 different kinds of physico-chemical properties. Some methods used these indices to train SVM and achieved a good performance [26]. Thus, we intend to create a logic sequential representation that takes into account properties of the Acid Index Database. Other points that we would like to explore are: the presence of short hydrophobic blocks in homologous proteins, as well as, structurally conserved amino acids [9], and functional amino acids, that is, active and binding sites. Moreover, we would like to replace WARMR with MineSeqLog [40]. MineSeqLog is an extension of WARMR that works on sequences where each sequence is an ordered list of ground predicates. This approach seems to be more suitable to deal with protein sequences, since the amino acid order is taken into account. PSI-BLAST performs better when run on nrdb90 with 20 iterations, and the use of PSI-BLAST output, as done in [24], to train our models provides another path to be explored.

## Methods

Here, we present our approach in detail. First, we describe the benchmark used for performing our experiments. Second, we present the first-order logical representations for protein sequences, this step is essential for using ILP systems. We present three kinds of logical representations. The first, named sequential, is based on properties coming directly from sequences. This representation being already proposed in previous works [31,32,35]. The second, named alignment, is based on conserved amino acid positions in a MSA. As the third representation, we related the first two representations creating a new one that takes into account conserved physico-chemical positions in a MSA. To the best of our knowledge the second and the third logical representations have been proposed here for the first time. Third, we present WARMR, the ILP system used here to learn logical rules, and explain how these rules are converted into binary attributes to train propositional models. Fourth, we describe the methodology used to assess and compare different methods. Finally, we discuss parameter settings and tools used in this work.

### Dataset description

In order to evaluate our methodology we used a common superfamily benchmark, that is SCOP database [41]. SCOP is a reference dataset for evaluating the performance of remote homology detection methods [9,16,21,22,24]. SCOP classifies all protein domains of known structure into a hierarchy with four levels: class, fold, superfamily, and family. In our study, we work at the superfamily level: it groups families for which a common evolutionary origin is not easily deduced from sequence identity, but rather from an analysis of structural and functional features. To provide a good comparability with previous approaches, we used the same database version used in [12,14,16,21,22,24]. It contains 54 families and 4352 proteins selected from SCOP version 1.53. All protein sequences were extracted from the Astral database [42] and all pairwise alignments have E-value no greater than 10^-25^.

We adopted the leave-one-family-out experimental methodology, as used in previous works. Thus, the sequences of each SCOP family are taken as positive test samples, and the proteins outside the family but within the same superfamily are taken as positive training samples. Negative samples are selected from outside of the superfamily and are separated into training and test sets. Previous works have considered random samples by splitting the *remaining *SCOP *database *(that is, SCOP minus the positive dataset) into training and test respecting the same ratio as the positive samples. This strategy produces unbalanced datasets: negative instances far outnumber the positive instances. For example, for the family test set "Nuclear receptor" in the "Glucocorticoid receptor-like (DNA-binding domain)" superfamily, there are 20 positive training sequences and 3204 negative training sequences. See the complete list with the distribution of positive and negative samples in Additional File 1 Table S1. If an unbalanced dataset is used to train a classifier, this latter will tend to predict that most of the incoming data belong to the majority class, that is the negative class. As a result, it would present poor predictive accuracy over the minority class, that is the positive one. To the best of our knowledge, previous works do not use a performance measure that evaluates the predictive accuracy over the minority class. They have used the area under the ROC curve (AUC-ROC) as performance measure, and AUC-ROC can present an excessively optimistic view on the algorithm performance when there is a large difference between positive and negative sample distributions [43]. We observed this behavior in Additional File 1 Table S2, where the AUC-ROC presents higher values than the area under the Precision-Recall curve (AUC-PR). Moreover, methods as "ILP-SVM-Seq" and "ILP-SVM-*Aln_cons_*", appear to be comparable in ROC Space, while in PR space, "ILP-SVM-*Aln_cons_*" has a clear advantage over "ILP-SVM-Seq".

Our analysis of protein sequence-identity in this unbalanced database, see Additional File 1 Figure S1-A, shows that around 46% of protein pairs have at least 30% of sequence-identity. Also, around 25% have sequence-identity between 90 and 100%. Moreover, we observed a bias in the composition of negative and positive classes: pairs of sequences in the negative set have, on average, higher sequence-identity than pairs of sequences in the positive set. This average is 22% for positive sequences, against 57% for negative sequences, see Additional File 1 Figure S1-B. We argue that this unbalanced database is not appropriate to evaluate the performance of remote homology detection methods, mainly because negative sequences are not into the *Twilight Zone*. Thus, we adopted a new experimental methodology to train and to test discriminative methods applied to the remote homology detection problem. The positive samples were taken as before, that is, within a SCOP superfamily. However, several negative samples were constructed by randomly selecting sets of sequences from the remaining SCOP database of size that is comparable to the size of the positive set. We constructed as many negative samples as it is needed to statistically cover the remaining SCOP database. For this, let *Tr*^+ ^and *Te*^+ ^be sizes of positive training and positive test sets, respectively. Also, let D* = *D *(*Tr*^+ ^+ *Te*^+^) be the size of the remaining SCOP database, where *D *is the total number of sequences in the SCOP database. Thus, we repeated the random selection of negative samples *T *times, where *T *is given by equation 1.(1)

In order to examine more systematically the performance of remote homology detection methods, we produced a database of sequences from the original one getting only sequences with identity smaller than 30%. It is named *S_30 _*and the original database *S_full_*. *S_30 _*contains 25 families and 2362 sequences.

### Logical representations

In order to use ILP systems, such as WARMR, first we have to represent each training and test examples as relational data. Good ILP overviews, including first-order logic concepts, can be found in [28,44,45]; here, we describe them briefly. We created three kinds of predicates, the first, called sequential predicates, represent each protein in terms of its physico-chemical properties and the frequency of their amino acids (taken alone or in pairs). The second, called alignment predicates, are based on conserved amino acid positions within a protein MSA. Additionally, we related both predicates to represent conserved physico-chemical positions within a protein MSA. The next sections explain them in detail.

#### First-order logic concepts

First-order logic, also called predicate logic, represents logic sentences in a more sophisticate way than the propositional logic. For example, consider the following sentences: "the amino acid *i *is hydrophobic" and "the amino acid *m *is hydrophobic". In propositional logic these sentences are treated as two unrelated propositions. On the other hand, the first-order logic can related them creating the predicate hydrophobic(*X*), which asserts that the amino acid represented by the variable *X *is hydrophobic. First-order logic allows us to define relation about properties that are shared among objects. For example, we can observed from Table 5 that a tiny amino acid is also a small amino acid. Then, we can denote this relation by using the logical rule *R_a_*: ∀(*X*)(tiny(*X*) → small(*X*)), where the symbol → is a logic connective used to denote a conditional (if/then) statement, and the symbol ∀ ("for all") is the universal quantifier symbol. The other quantifier is the ∃ ("there exists") called existential quantifier. The part of *R_a _*before connective → is called antecedent and the part after is called consequent. The standard logic connectives are ⋀ for conjunction, ⋁ for dis-junction, → for implication, ↔ for bi-conditional and ¬ for negation. Next, we define some syntax rules for the first-order logic language. A variable (*X*, *Y *and *W *in Table 5) is a term. If *t*_1, _..., *t_n _*are terms and *f *is a function symbol then *f*(*t*_1_, ..., *t_n_*) is a term, where *n *is arity (number of arguments) of the function, and *n *≥ 0. A function of zero arity (*n *= 0) is a constant (*c*, *k*, *cg*, 24, 27, ... in Table 1). If *t*_1_, ..., *t_n _*are terms and *p *is a (*n *≥ 0) predicate symbol then *p*(*t*_1_, ..., *t_n_*) is an atomic formula, also called here predicate. More complex formulae can be built using the logical connectives and quantifiers. A substitution *s *= *X*/*i *is an assignment of term *i *to variable *X*. For example, when s is applied to the predicate hydrophobic(*X*) an instantiation of the predicate hydrophobic(*i*) is created. A ground predicate is a predicate without any variables.

**Table 5:** Sequential Predicates

Property/amino acid set	Predicate
1- small {A,G,S,T}	small(X,Y)

2- polar {D,E,H,K,N,Q,R,S,T,W,Y}	polar(X,Y)

3- polar uncharged {N,Q}	polarUncharged(X,Y)

4- aromatic {F,H,W,Y}	aromatic(X,Y)

5- charged {D,E,H,I,K,L,R,V}	charged(X,Y)

6- positively charged {H,K,R}	positivelyCharged(X,Y)

7- negatively charged {D,E}	negativelyCharged(X,Y)

8- tiny {A,G}	tiny(X,Y)

9- bulky {F,H,R,W,Y}	bulky(X,Y)

10- aliphatic {I,L,V}	aliphatic(X,Y)

11- hydrophobic {I,L,M,V}	hydrophobic(X,Y)

12- hydrophilic basic {K,R,H}	hydrophilicBasic(X,Y)

13- hydrophilic acidic {E,D,N,Q}	hydrophilicAcidic(X,Y)

14- neutral weakly hydrophobic {A,G,P,S,T}	neutralWeakHydrophobic(X,Y)

15- hydrophobic aromatic {F,W,Y}	hydrophobicAromatic(X,Y)

16- acidic {E,D}	acidic(X,Y)

17- amino acid ratio	aminoacidRatio(X,W,Y)

18- amino acid pair ratio	aminoacidPairRatio(X,W,Y)

For each predicate like *property*(*X*, *Y*) numbered from 1 to 18, *X *is the sequence identifier, *Y *is the percentage of amino acids with some physico-chemical property. For the predicate *aminoacidRatio*(*X*, *W*, *Y*), *X *is the sequence identifier, W is an amino acid, and *Y *is the percentage of amino acid W within sequence *X*. For the predicate *aminoacidPairRatio*(*X*, *W*, *Y*), *X *and *Y *are defined as before, and *W *is a pair of amino acids.

#### Sequential predicates

The sequential predicates are based on properties that can be calculated directly from sequences. These include groups of amino acids that share some physico-chemical properties as done in [35], see Table 5 from property 1 to 16. Additionally, we created predicates to represent the distribution for singles and pairs of residues as done in [29-32], showed in Table 5 properties 17 and 18. All predicates used in this study are listed in Table 5. The variable *Y *can assume only numerical values, however ILP systems such as WARMR are not very suitable for handing with numerical values. To overcome this limitation we map each percentage value *Y *to ⌊*Y*/*10*⌋ + *1*, as done in [31,32].

#### Alignment predicates

**Figure 4:**
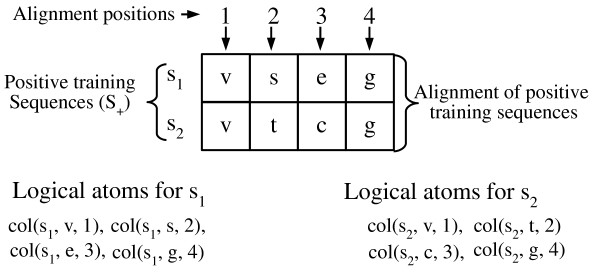
**Creating ground predicates from alignment positions**. A ground predicate is defined for each alignment position. For instance, the ground predicate *col*(*s_1_*, *v*, *1*) means that the sequence *s_1 _*has the amino acid *v *in the first alignment position.

Additionally, we created a predicate based on conserved positions in a protein MSA. The predicate that represents each alignment position is *col*(*X*, *W*, *Z*), where *X *is the sequence identifier, *Z *is the alignment position where the amino acid *W *belongs. To illustrate how these predicates are created, see Figure 4. The sequences in the positive training set (*S*_+_) are aligned and a ground predicate is created for each amino acid in each alignment position. For example, the ground predicate *col*(*s_1_*, *v*, *1*) means that the sequence *s_1 _*has the amino acid *v *in the first alignment position.

The creation of these ground predicates for the sequences of the positive training set is a trivial task, since they can be extracted directly from the MSA. However, how can we create ground predicates for the query sequences? We aim to find out how a query sequence is aligned in respect to the positive training alignment (homologous proteins). If the query sequence is closely matched to the positive training alignment, probably this suggests much higher conservation than for a query sequence weakly matched. Therefore, we must x the positive training alignment and align a query sequence against it, that is, we aligned each query sequence with the consensus sequence of the positive training alignment. To do this, we built a pHMM from the positive training alignment, and used it for matching the query sequences.

**Figure 5:**
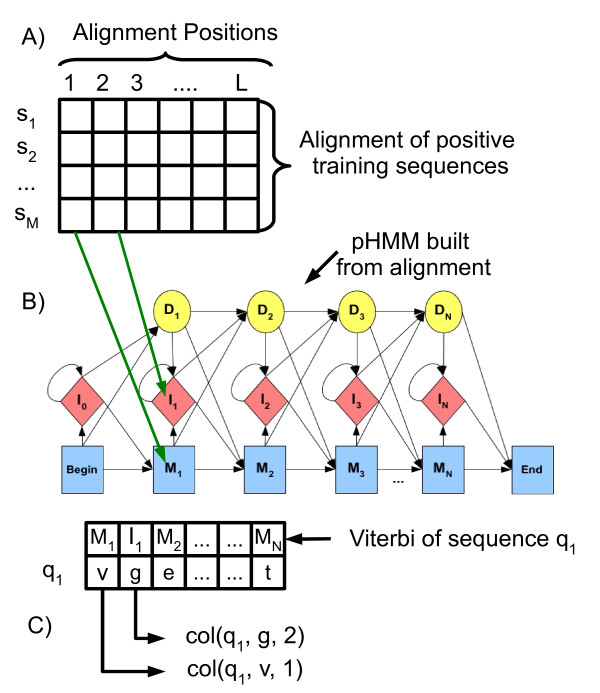
**Using pHMMs to create ground predicates for alignment positions**. A) First, the positive training sequences are aligned. B) Second, a pHMM is built from this alignment. Each query sequence (negative training sequences, and positive and negative test sequences) is matched by pHMM producing the Viterbi output that shows the correspondence between each amino acid in the query sequence and pHMM states (match (M), insert (I) or delete (D)). C) Finally, we know the mapping between alignment positions and pHMM states, thus we create, for each query sequence, ground predicates in a similar way to Figure 4.

A pHMM represents an alignment of homologous sequences by creating a sequence of nodes, where each node is composed of three states: match (M), insert (I) and delete (D), for example, {*M*_1_, *I*_1_, *D*_1_} in architecture of Figure 5. Match states model conserved regions in the alignment, and insert and delete states model *indel *regions. During the training phase each alignment position is mapped to a node, and parameters of the model are estimated. Next, inference algorithms like Viterbi are used to match a query sequence against the model.

The Viterbi algorithm also gives us the pHMM's state sequence that better has recognized the query sequence, that is, it maps each amino acid in the query sequence to a match (M), insert (I) or delete (D) states. Since, each alignment position corresponds to a pHMM state, we can determine how the query sequence was aligned in respect to the positive training alignment. The schema of Figure 5 shows how to create ground predicates based on alignment positions for negative training sequences, and for positive and negative test sequences.

#### Relating sequential and alignment predicates: conserved physico-chemical position in a MSA

Through first-order logic, new knowledge statements can be extracted from data relations. For example, observe the position 34 in the alignment shown in Figure 2. All amino acids in this position are small (see Table 5) thus, we can learn a logical rule that relates position 34 to small amino acids (see *R*_6 _in Table 1). This rule allows us to introduce the new concept of "conserved physico-chemical position" in a MSA.

### Construction of propositional classifiers

In our approach we aim to build models that will be able to explore the most frequent patterns in the homologous protein datasets. As a first step we run WARMR program to learn these most frequent patterns on the positive training set. The WARMR algorithm discovers frequent patterns on databases applying an extended version of APRIORI algorithm [46]. WARMR learns association rules over multiple relations in relational datasets. Basically, WARMR algorithm works as a filter on all possible rules selecting, for example, those rules with *confidence *above a threshold. The confidence of an association rule is a percentage value that shows how frequently the rule occurs. In other words, the confidence value indicates how reliable this rule is. As a second step (propositionalization step), we converted each rule learned by WARMR into a binary attribute (feature) for the training of propositional learning methods. An attribute *a_i _*has value *1 *for a specific protein sequence if the corresponding query *r_i _*succeeds, and *0 *if the query fails. Finally, we trained two propositional models from these attributes, DTs and SVMs.

### Comparison between different methods

To statistically analyse remote homology detection methods, we run them several times over the same positive set, but over several negative sets. In each run the number of positive samples is equal to the number of negative samples. Since datasets are now balanced curves in the AUC-PR space are similar to curves in the AUC-ROC space [43]. Therefore, we just show AUC-ROC as the classification accuracy measure. For each protein family, the AUC-ROC score was averaged over *T *runs (see equation 1), and the overall performance of each method was averaged over all families.

### Parameter settings and tools used

We used CLUSTALW [47] version 2.0.10 in order to provide the positive training alignments. The CLUSTALW parameters were kept at default. We used HMMer [48] version 2.3.2 (default parameters) to build the pHMMs from positive training alignments. These pHMMs were used to score query sequences and their output was used to construct logical representations based on alignment positions. In order to learn logical rules we used WARMR. The confidence parameter (c%) of the WARMR filters the most frequent patterns, that is, only those with frequency above c% are considered. We tested several threshold values for c% and the best results were obtained with 25% for logical representations based on sequential properties and based on conserved amino acid positions, and 50% for the representation based on conserved physico-chemical positions.

Next, the rules generated by these representations were converted into binary attributes for training propositional models. We have created two kinds of propositional models: DT and SVM. For SVM we have used the publicly available Gist SVM package version 2.1.1 http://svm.sdsc.edu. We used radius basis function as kernel function and other parameters by default, and DT models were built using the WEKA software (default parameters) available in http://www.cs.waikato.ac.nz/~ml/weka/index_downloading.html. In order to compare our approach with state of the art methods, we consider SVM-LA [16], SVM-Ngram-LSA [22], PSI-BLAST and HMMer-3.0. SVM-LA is a complex method kernel that defines a similarity measure between protein pairs by summing up scores obtained from their local alignment. The SVM-LA parameters were kept as default. SVM-Ngram-LSA extracts N-gram from protein sequences and uses them to train a SVM model. To consider only the most significant N-grams it applies Latent Semantic Analysis (LSA), which is a feature extraction technique from natural language processing. We downloaded SVM-Ngram-LSA from http://www.insun.hit.edu.cn/news/view.asp?id=413 and used it with parameters described in [22]. HMMer-3.0 was trained from MSAs produced by CLUSTALW, and all parameters were kept as default. PSI-BLAST was ran on two configurations: in the first, we used the same dataset used to train the other methods and 4 iterations; in the second we used nrdb90 and 20 iterations. We also considered to compare to Top-N-gram [24], a recent work that applies SVM to the remote homology detection problem. However, the program was unavailable. We used chi-square as a feature selection approach, and we set the parameter *δ *to 0.05 and 0.25 values, as done in [49], *δ *specifies the confidence level for the chi-square test selection. We carried out rank-sum test [38] to compare the curves showed in Figure 3.

## Availability and Requirements

The software is available upon request. It was implemented in JAVA and perl and works on linux plataform. WARMR and GIST are required to run our system.

## Authors' contributions

JSB implemented the system, performed the experiments and drafted the manuscript. AC designed the experiments, contributed to the analysis of the results, and helped writing the manuscript. GZ designed and supervised the project and contributed to the writing of the manuscript. All authors read and approved the final manuscript.

## Supplementary Material

Additional file 1 **Analysis and characteristics of original unbalanced database**. We carried out an analysis on the original dataset, that is, the database communally used to evaluate the performance of the state of art methods. Click here for file
