# Supplementary material for: A discriminative method for family-based protein remote homology detection that combines inductive logic programming and propositional models
Source: BMC Bioinformatics. 2011 Mar 23;12:83. doi: 10.1186/1471-2105-12-83 (PMC3078102; doi:10.1186/1471-2105-12-83)
Supplement: Additional file 1 — Analysis and characteristics of original unbalanced database. We carried out an analysis on the original dataset, that is, the database communally used to evaluate the performance of the state of art methods. [file 1471-2105-12-83-S1.PDF]

Additional File 1

Juliana S. Bernardes, Alessandra Carbone and Gerson Zaverucha

March 7, 2011

List of Tables

1 **Distribution of positive and negative samples for the original unbalanced database**. Tr^+, Te^+, Tr^- and Te^- are sizes of positive training, positive test, negative training and negative test sets, respectively. 2
2 **AUC-ROC and AUC-PR for ILP-SVM models trained from the original unbalanced database.** 2

List of Figures

1 **Analysis of sequence-identity for the original unbalanced database.** A) Distribution of sequence-identity for pairs of proteins for the whole database. On the left, we show all sequence-identity distribution, and on the right, we highligth two groups, that is, 0-30% and 30-100%. B) Distribution of sequence-identity for pairs of protein for the positive and negative sets. On the left, we show the sequence-identity distribution for protein pairs in the positive set, and on the right for protein pairs in the negative set. 3

Table 1: **Distribution of positive and negative samples for the original unbalanced database**. Tr^+, Te^+, Tr^- and Te^- are sizes of positive training, positive test, negative training and negative test sets, respectively.

SCOP Family	Tr^+	Tr^-	Te^+	Tr^-
7.3.5.2	12	2330	9	1746
2.56.1.2	11	2509	8	1824
3.1.8.1	19	3002	8	1263
3.1.8.3	17	2686	10	1579
1.27.1.1	12	2890	6	1444
1.27.1.2	10	2408	8	1926
3.42.1.1	29	3208	10	1105
1.45.1.2	33	3650	6	663
1.4.1.1	26	2256	23	1994
2.9.1.2	17	2370	14	1951
1.4.1.2	41	3557	8	693
2.9.1.3	26	3625	5	696
1.4.1.3	40	3470	9	780
2.44.1.2	11	307	140	3894
2.9.1.4	21	2928	10	1393
3.42.1.5	26	2876	13	1437
3.2.1.2	37	3002	16	1297
3.42.1.8	34	3761	5	552
3.2.1.3	44	3569	9	730
3.2.1.4	46	3732	7	567
3.2.1.5	46	3732	7	567
3.2.1.6	48	3894	5	405
2.28.1.1	18	1246	44	3044
3.3.1.2	22	3280	7	1043
3.2.1.7	48	3894	5	405
2.28.1.3	56	3875	6	415
3.3.1.5	13	1938	16	2385
7.3.10.1	11	423	95	3653
3.32.1.11	46	3880	5	421
3.32.1.13	43	3627	8	674
7.3.6.1	33	3203	9	873
7.3.6.2	16	1553	26	2523
7.3.6.4	37	3591	5	485
2.38.4.1	30	3682	5	613
2.1.1.1	90	3102	31	1068
2.1.1.2	99	3412	22	758
3.32.1.1	42	3542	9	759
2.38.4.3	24	2946	11	1349
2.1.1.3	113	3895	8	275
2.1.1.4	88	3033	33	1137
2.38.4.5	26	3191	9	1104
2.1.1.5	94	3240	27	930
7.39.1.2	20	3204	7	1121
2.52.1.2	12	3060	5	1275
7.39.1.3	13	2083	14	2242
1.36.1.2	29	3477	7	839
3.32.1.8	40	3374	11	927
1.36.1.5	10	1199	26	3117
7.41.5.1	10	2241	9	2016
7.41.5.2	10	2241	9	2016
1.41.1.2	36	3692	6	615
2.5.1.1	13	2345	11	1983
2.5.1.3	14	2525	10	1803
1.41.1.5	17	1744	25	2563

Table 2: **AUC-ROC and AUC-PR for ILP-SVM models trained from the original unbalanced database.**

	AUC-ROC	AUC-PR
ILP-SVM-Seq	0.81	0.10
ILP-SVM-Aln_{cons}	0.83	0.21
ILP-SVM-Seq-Aln_{cons}	0.85	0.22

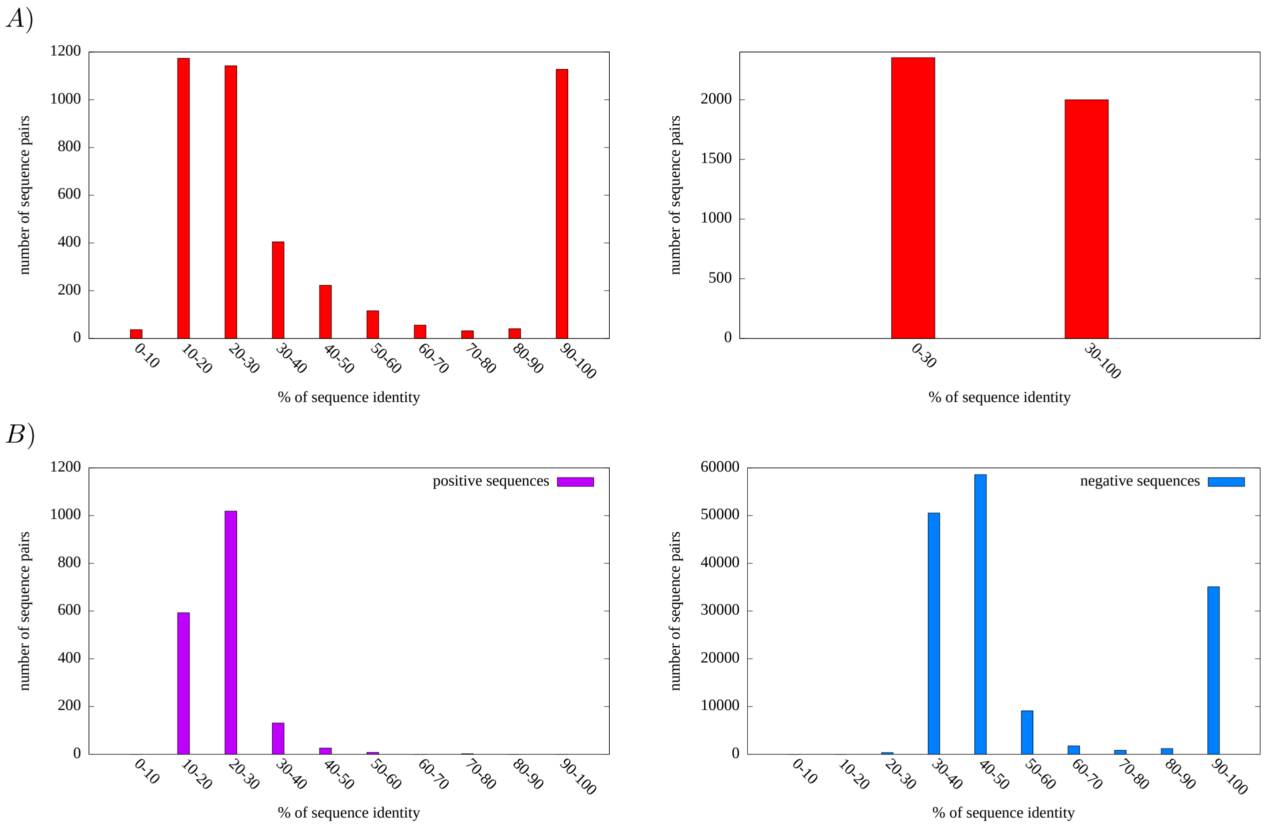

Figure 1: **Analysis of sequence-identity for the original unbalanced database.** A) Distribution of sequence-identity for pairs of proteins for the whole database. On the left, we show all sequence-identity distribution, and on the right, we highligth two groups, that is, 0-30% and 30-100%. B) Distribution of sequence-identity for pairs of protein for the positive and negative sets. On the left, we show the sequence-identity distribution for protein pairs in the positive set, and on the right for protein pairs in the negative set.
